# Quantitative Spatial Analysis of Neuroligin-3 mRNA Expression in the Enteric Nervous System Reveals a Potential Role in Neuronal–Glial Synapses and Reduced Expression in *Nlgn3^R451C^* Mice

**DOI:** 10.3390/biom13071063

**Published:** 2023-06-30

**Authors:** Madushani Herath, Ellie Cho, Ulrika Marklund, Ashley E. Franks, Joel C. Bornstein, Elisa L. Hill-Yardin

**Affiliations:** 1 Department of Anatomy & Physiology, University of Melbourne, Parkville, VIC 3010, Australia; shani.maduwelthanneherathmud@bcm.edu (M.H.); j.bornstein@unimelb.edu.au (J.C.B.); 2 Biological Optical Microscopy Platform, University of Melbourne, Parkville, VIC 3010, Australia; ellie.cho@unimelb.edu.au; 3 Division of Molecular Neurobiology, Department of Medical Biochemistry and Biophysics, Karolinska Institute, 171 77 Stockholm, Sweden; ulrika.marklund@ki.se; 4 Department of Microbiology, Anatomy Physiology & Pharmacology, School of Agriculture, Biomedicine and Environment, La Trobe University, Bundoora, VIC 3086, Australia; a.franks@latrobe.edu.au; 5 School of Health and Biomedical Sciences, STEM College, RMIT University, Bundoora, VIC 3083, Australia

**Keywords:** neuroligin-3, autism, enteric nervous system, glia, ileum, RNAScope, immunocytochemistry, scRNASeq

## Abstract

Mutations in the Neuroligin-3 (*Nlgn3*) gene are implicated in autism spectrum disorder (ASD) and gastrointestinal (GI) dysfunction, but cellular *Nlgn3* expression in the enteric nervous system remains to be characterised. We combined RNAScope in situ hybridization and immunofluorescence to measure *Nlgn3* mRNA expression in cholinergic and VIP-expressing submucosal neurons, nitrergic and calretinin-containing myenteric neurons and glial cells in both WT and *Nlgn3^R451C^* mutant mice. We measured *Nlgn3* mRNA neuronal and glial expression via quantitative three-dimensional image analysis. To validate dual RNAScope/immunofluorescence data, we interrogated available single-cell RNA sequencing (scRNASeq) data to assess for *Nlgn3*, *Nlgn1*, *Nlgn2* and their binding partners, *Nrxn1-3*, *MGDA1* and *MGDA2*, in enteric neural subsets. Most submucosal and myenteric neurons expressed *Nlgn3* mRNA. In contrast to other *Nlgns* and binding partners, *Nlgn3* was strongly expressed in enteric glia, suggesting a role for neuroligin-3 in mediating enteric neuron–glia interactions. The autism-associated R451C mutation reduces *Nlgn3* mRNA expression in cholinergic but not in VIPergic submucosal neurons. In the myenteric plexus, *Nlgn3* mRNA levels are reduced in calretinin, nNOS-labelled neurons and S100 β -labelled glia. We provide a comprehensive cellular profile for neuroligin-3 expression in ileal neuronal subpopulations of mice expressing the R451C autism-associated mutation in *Nlgn3,* which may contribute to the understanding of the pathophysiology of GI dysfunction in ASD.

## 1. Introduction

Neuroligin-3 (NLGN3) is an adhesion molecule expressed at neuronal synapses in the central nervous system (CNS) and is well established as a regulator of brain function [1,2,3,4,5]. Although some studies suggest that NLGN3 is important for proper GI function [6,7], its cellular expression profile in the enteric nervous system has not been characterised. In cultured rat hippocampal neurons, NLGN3 is expressed at the postsynaptic membrane of both excitatory and inhibitory neuronal synapses [8]. NLGN3 is also expressed in non-neuronal cells. For example, Venkatesh and colleagues showed that neurally secreted NLGN3 invades the microenvironment of tumours and induces NLGN3 expression in glioma cells to promote tumour growth [9]. In addition, NLGN3 is expressed in many types of glia during rodent development, including olfactory ensheathing glia, retinal astrocytes, Schwann cells and spinal cord astrocytes [10]. These expression patterns suggest that NLGN3 could also be expressed in enteric glia.

Mutations in the *NLGN3* gene, including the missense R451C point mutation as well as *NLGN3* deletion, are implicated in autism [11,12,13]. Patients expressing the *NLGN3* R451C mutation show GI dysfunction, including diarrhoea, faecal incontinence, post-meal regurgitation, oesophageal inflammation, chronic intestinal pain as well as delayed bladder and bowel control [7]. Modification of NLGN3 expression induces GI dysfunction in mice, as shown in both *Nlgn3* knockout (KO) and *Nlgn3^R451C^* mice [6,7]. *Nlgn3* KO mice exhibit distended colons and more rapidly propagating colonic muscle contractions [6]. *Nlgn3^R451C^* mice display faster intestinal transit and increased numbers of myenteric neurons in the small intestine as well as GABA_A_ receptor-mediated colonic dysmotility [7]. These findings suggest a role for NLGN3 in gut function.

The R451C mutation impacts *Nlgn3* mRNA and protein expression in the brain. In mouse whole-brain samples, *Nlgn3* mRNA expression levels are unchanged by the *Nlgn3* R451C mutation, but NLGN3 protein levels are reduced [1]. Although initial studies of NLGN3 expression in the GI tract have been reported in rodents [7,14,15] and humans [14], the distribution of NLGN3 expression in different cell populations of the mouse enteric nervous system has not been profiled. Therefore, the objective of this study was to provide comprehensive cellular profiling for neuroligin-3 expression in subpopulations of mouse myenteric and submucosal neurons using RNAScope, RNASeq data mining and immunofluorescence analyses. We also assessed for changes in neurochemical markers and *Nlgn3* gene expression in the enteric nervous system of mice expressing the R451C autism-associated mutation in *Nlgn3*. Characterising *Nlgn3* expression in enteric cell subtypes is essential for identifying underlying mechanisms contributing to GI activity and to functional GI changes observed in patients and the *Nlgn3^R451C^* mouse model of autism. 

## 2. Materials and Methods

### 2.1. Animals 

B6;129-Nlgn3tm1Sud/J mice were obtained from The Jackson Laboratory [1] and bred for over 10 generations on a C57BL/6 background at the Howard Florey Institute, Melbourne, Australia. Mice were subsequently housed in the Biomedical Science Animal Facility at The University of Melbourne. Male *Nlgn3^R451C^* and wild-type (WT) mice (age 12–13 weeks) were sacrificed by cervical dislocation in accordance with The University of Melbourne Animal Experimentation Ethics Committee (#1914843). For each set of neuronal markers analysed, a different group of WT and mutant mice were used. In total, 68 mice were used in the study. These numbers are noted for each experimental component within the results section and supplementary information Table S1 (63 mice in total for RNAscope; 34 WT and 29 *Nlgn3*^R451C^ mutant mice). In addition, 5 mice were included in the RNAseq study reported by Morarach et al. in 2021 [16].

### 2.2. Tissue Preparation 

Mice were sacrificed, and the abdomen was opened using coarse dissection scissors. A 2 cm segment of the distal ileum (1 cm proximal to the caecum) was isolated and immediately placed in oxygenated ice-cold physiological saline solution (composition in mM: 118 NaCl, 4.6 KCl, 2.5 CaCl_2_, 1.2 MgSO_4_, 1 NaH_2_PO_4_, 25 NaHCO_3_, 11 D-glucose). The intestinal content was gently flushed clean using a Pasteur pipette. Using small dissecting scissors, the distal ileal tissue sample was cut along the mesenteric border and then stretched and pinned flat on a chilled silicon elastomer-lined dish (Sylgard 184, Dow Corning, Pennant Hills, NSW, Australia). The tissue was fixed in 4% formaldehyde for 24 h at 4 °C. After fixation, the tissues were washed three times in 1M phosphate buffer saline (PBS) (composition in mM: 137 NaCl, 2.7 KCl, 8 Na_2_HPO_4_, 2 KH_2_PO_4_). The wholemount submucosal plexus preparation was isolated by peeling the mucosa–submucosal layer and then by removing the epithelium using fine forceps (Fine Science Tools, North Vancouver, CO, Canada). Myenteric plexus preparations including the longitudinal muscle layer (LMMP) were obtained by microdissection to remove the circular muscle layer. 

### 2.3. Dual RNAScope In Situ Hybridization and Immunofluorescence 

Localizing the NLGN3 protein in the mouse GI tract is challenging due to non-specific labelling of commercially available antibodies which commonly yield false-positive results in this tissue (Leembruggen et al., unpublished). Therefore, we used the enhanced in situ hybridization technique, RNAScope, to label *Nlgn3* mRNA in enteric neurons. We combined RNAScope and immunofluorescence with high-resolution microscopy and 3D image analysis software to compare *Nlgn3* mRNA expression in the wild-type (WT) and *Nlgn3^R451C^* mouse gut. 

Using RNAScope, individual RNA molecules are visualized using a novel probe design and amplification system to simultaneously magnify the signal and suppress background noise [17]. RNAScope in situ hybridization in mouse ileal tissue was performed using the ACDBio multiplex RNAScope assay kit (ACD, Newark, CA, USA) according to manufacturer’s instructions. Briefly, wholemount mouse ileal submucosal and LMMP preparations were pre-treated and hybridized with the RNAScope probe of interest. The hybridization signal was subsequently amplified via a sequence of amplifiers and fluorescently labelled probes. 

#### 2.3.1. Tissue Pre-Treatment 

Wholemount preparations of submucosal and LMMP preparations were permeabilized via pre-treatment with protease IV prior to RNAScope in situ hybridisation. The tissue samples were incubated in 30 μL of protease IV for 30 min at room temperature (RT) in a sealed humid chamber and washed twice (1 min in each) in 1 M PBS. 

#### 2.3.2. RNAscope In Situ Hybridization 

Following pre-treatment, ileal tissue preparations were incubated in 20 μL of *Nlgn3* RNAScope probe and incubated for 2 h at 40 °C in a humid chamber. The ileal tissues were washed twice in 1× wash buffer for 2 min at RT with agitation. Amplification and detection steps were performed using the RNAScope detection kit reagents. For the signal amplification step, tissues were incubated in 50 μL of amplifier 1 solution (AMP 1) for 30 min at 40 °C and washed twice in 1× wash buffer for 2 min at RT with occasional agitation. Subsequently, the tissue was incubated in 50 μL of AMP 2 solution for 15 min at 40 °C and then washed twice in 1× wash buffer for 2 min at RT with occasional agitation. After AMP 2 amplification, 50 μL of AMP 3 solution was added to the tissue preparations and incubated for 30 min at 40 °C. The tissue was washed twice in 1× wash buffer for 2 min at RT with occasional agitation. Lastly, the tissue was incubated in 50 μL of AMP 4 solution and incubated for 15 min at 40 °C prior to washing the tissue twice in 1× wash buffer for 2 min at RT with occasional agitation. The same protocol was followed for control experiments using the RNAScope universal negative control dapB probe (Supplementary Figure S1).

#### 2.3.3. Immunofluorescence

After washing three times in PBS, the wholemount preparations were permeabilised with 1% Triton X-100 (ProSciTech, Kirwan, Australia) for 30 min at RT and washed three times in PBS. The tissues were then double labelled with various combinations of primary antibodies at different incubation conditions. Excess primary antibodies were removed by washing in PBS (3 × 10 min), and the tissue samples were subsequently incubated in secondary antibodies for 2 h at 4 °C. After removing excess antisera by washing with 0.1 M PBS (3 × 10 min), tissue preparations were mounted using Dakocytomation fluorescence mounting medium (DAKO; Carpinteria, CA, USA). The primary and secondary antibodies that were used to label these neuronal subpopulations and glial cells are listed in Table 1 and Table 2, respectively. The number of animals used in each experiment and for different markers are included in the results section and supplementary information Table S1. 

#### 2.3.4. Submucosal Plexus Antibodies

Given that the submucosal plexus is predominantly involved in regulating secretory function in the GI tract, we endeavoured to study functionally relevant cell types in this region. It is well established that the two main groups of submucosal secretomotor neurons are cholinergic (i.e., expressing ChAT) and non-cholinergic (and expressing VIP) secretomotor neurons (e.g., Foong et al., 2014 [18]). Therefore, we utilised ChAT and VIP antisera to identify these prominent functional subtypes using immunofluorescence, in combination with RNAScope for *Nlgn3* expression.

#### 2.3.5. Myenteric Plexus Antibodies

Similarly, in the myenteric plexus, we aimed to study major functional neuronal subtypes. For example, inhibitory motor neurons and a subpopulation of descending interneurons in the myenteric plexus express nitric oxide; therefore, we used neuronal NOS (nNOS) antiserum to investigate the distribution of *Nlgn3* in these neurons. In contrast, only 3% of neurons in the submucosal plexus are labelled with nNOS [19]; thus, the nNOS antiserum was not utilized in the submucosal plexus in the current study. In the myenteric plexus, calretinin is expressed in many different neuronal subtypes, including excitatory motor neurons, ascending interneurons, intrinsic sensory neurons [20]. In addition, calretinin is a useful marker in the myenteric plexus given it has been demonstrated that there is minimal co-expression between nNOS and calretinin in mouse small intestinal myenteric neurons (Sang and Young, 1996). Although calretinin is also expressed in non-cholinergic submucosal plexus neurons, we used VIP staining in the submucosal plexus to identify this neuronal group. 

In the small intestine, Sang and Young [21] also found that almost all NOS neuronal cell bodies also contained VIP. For this reason, we utilized NOS to identify a major proportion of neurons in the myenteric plexus, but not VIP staining. In addition, VIP labelling of neuronal cell bodies in the myenteric plexus is unreliable. The ChAT antiserum was not utilized in the myenteric plexus given that this neuronal group corresponds to cells that do not express NOS (i.e., more than 90% of non-NOS labelled myenteric neurons in the mouse small intestine are known to contain ChAT [21]); therefore, the use of ChAT in the myenteric plexus would not have yielded additional information regarding neuron types. 

### 2.4. Image Acquisition and Analysis 

Multi-channel image acquisition was performed with a laser scanning confocal microscope 800 (Carl Zeiss Microscopy, North Ryde, NSW, Australia) using a 40× oil immersion objective lens. Tissue samples were excited with diode lasers at 488, 561 and 647 wavelengths. Pinhole diameter, detector gain and laser power parameters were optimised to obtain the highest pixel intensity at the same time eliminating pixel saturation. Consecutive Z stacks on the horizontal plane with a frame size of 1024 × 1024 pixels and a bit-depth of 16 bits were captured at 1 µm intervals. Z-stacks together with tile scans were performed where necessary. 

Quantification of labelling for RNAscope puncta and antisera was performed using 3D reconstruction tools and statistical annotation for quantification purposes in Imaris 9.0 image analysis software (Bitplane, Zurich, Switzerland). Three-dimensional cell rendering was undertaken using high-resolution Z-stack fluorescent images generated using confocal microscopy. To create a detailed rendered surface, the “Surface” tool in the Imaris software package was used. The minimum diameter of the surface was determined based on the average cell diameter estimated using the “Slice view”. The interactive software histogram within the “Create surface” window was used to set a threshold (the minimum diameter of the neuronal surface) to exclusively include neuronal surfaces and to exclude smaller surfaces generated by the background noise. Aggregated neurons within images were separated using the ‘Split surfaces” tool in Imaris. The Imaris software is additionally equipped with “Filter options” to label specific populations of surfaces either manually or automatically, and these were utilized in the current study to identify specific cell populations in any given image. Since the RNAScope signal representing *Nlgn3* mRNA appears as puncta, the “spot” analysis tool in Imaris was used to create the 3D structure of individual mRNA molecules. The “average diameter of spots” detected was determined using “slice view” in Imaris. Once spots were generated, they were assigned as being located within the neurons using the “Split spots on to surface” tool. The Imaris 9.0 software additionally generates statistical data yielding the number of spots per surface. This option was used to identify the number of mRNA punctate signals within the neurons for the images analysed in the current study. 

### 2.5. Statistical Analysis

Data were analysed using GraphPad Prism 8.4.1 (GraphPad software, San Diego, CA, USA). A different group of animals was used to investigate labelling for each set of enteric cellular subpopulations. Frequency distribution analysis was performed to analyse the distribution of *Nlgn3* mRNA expression in enteric neurons and glia, and a two-sample Kolmogorov–Smirnov test was conducted to determine statistical significance. In general, to estimate the extent of overlap between two distributions for *Nlgn3* mRNA expression between genotypes, the magnitude of the Kolmogorov–Smirnov D statistic (distance; D) relative to the critical value is obtained. D is a measure of the maximum distance between the empirical cumulative distribution function of the sample and the cumulative distribution function of the reference distribution (if the value of D is larger than the critical value, the null hypothesis is rejected). Analysis using the GraphPad Prism algorithm provides a *p* value calculated based on the maximum distance between the cumulative frequency distributions, considering both D and the D critical value. 

### 2.6. Investigation of Nlgn, Nrxn and MDGA Gene Family Expression in Single-Cell RNA Sequencing Datasets

To further validate the RNAScope findings in the current study, we analysed published scRNA sequencing data identifying molecular and functional subtypes of enteric neurons in mice [16] (http://ncbi.nlm.nih.gov/sra/SRP258962; URL accessed on 4 September 2022) and Zeisel et al. [22] (https://www.ncbi.nlm.nih.gov/sra/SRP135960; URL accessed on 4 September 2022). ScRNASeq was performed on isolated neurons from myenteric peels of mice aged postnatal day (P)21, and marker genes for neuron types were validated using immunofluorescence in the ENS of P21-90 mice [16]. The transcriptome of single enteric glia cells isolated from P21 and P23 myenteric plexus was assessed using mousebrain.org (web interface for browsing data presented in Zeisel et al., 2018 [22]).

## 3. Results

Here, we characterized the cellular localization of *Nlgn3* mRNA in the enteric nervous system of the mouse distal ileum. Using RNAscope and immunofluorescence in WT mice, we first determined the frequency distribution and the average number of *Nlgn3* mRNA copies per cell in neuronal subpopulations and glia. Using scRNASeq datasets, we then identified enteric neuronal subsets expressing *Nlgn3* and their binding partners, the neurexins (*Nrxns1*, *2* and *3*) and MAM domain-containing glycosylphosphatidylinositol anchor genes (*MDGA1* and *2*). Subsequently, we investigated whether the R451C missense mutation in *Nlgn3* impacts cellular expression profiles in the enteric nervous system of the mouse ileum using RNAscope and immunofluorescence. 

### 3.1. Nlgn3 mRNA Is Expressed in Most Distal Ileal Submucosal Neurons 

**Figure 1:**
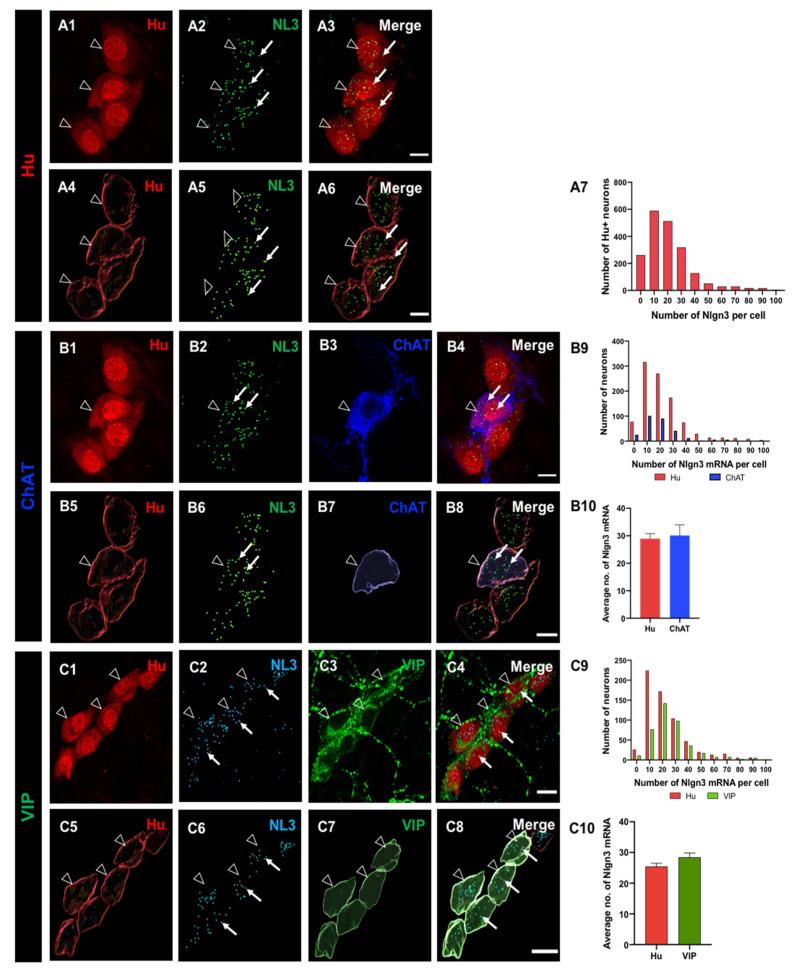
The distribution of *Nlgn3* mRNA in ileal submucosal neurons. Confocal images of (**A1**) submucosal neurons labelled with Hu pan neuronal marker; (**A2**) *Nlgn3* mRNA expression; (**A3**) merged image; (**A4**) 3D reconstruction of submucosal neurons; (**A5**) cellular *Nlgn3* mRNA expression; (**A6**) merged image; (**A7**) frequency distribution of *Nlgn3* mRNA expression in submucosal neurons. Confocal images of (**B1**) submucosal ganglion (**B2**) *Nlgn3* mRNA expression, (**B3**) ChAT neuron, (**B4**) merge; 3D structure of (**B5**) submucosal neurons, (**B6**) *Nlgn3* mRNA, (**B7**) a cholinergic submucosal neuron (**B8**), merge; (**B9**) distribution of *Nlgn3* mRNA expression in cholinergic neurons is similar to that in total neurons in the submucosal plexus; (**B10**) average number of *Nlgn3* mRNA copies in cholinergic neuronal soma is similar to that in total neurons in the submucosal plexus. Confocal micrographs of: (**C1**) submucosal neurons, (**C2**) *Nlgn3* mRNA expression, (**C3**) VIP expression, (**C4**) merge; 3D reconstruction of (**C5**) submucosal neurons, (**C6**) *Nlgn3* mRNA expression (**C7**) VIP-expressing neurons; (**C8**) merge; (**C9**) frequency distribution of *Nlgn3* mRNA expression in VIP submucosal neurons is significantly different to total submucosal neurons; (**C10**) on average, VIP submucosal neurons express similar numbers of *Nlgn3* mRNA copies as observed in the total number of neurons in the submucosal plexus. Open arrowheads: Hu staining; arrows: *Nlgn3* mRNA expression. Scale bar = 10 µm.

All submucosal neurons in distal ileal preparations were labelled with the pan-neuronal marker Hu. We then analysed the distribution of *Nlgn3* mRNA expression in somata of individual submucosal neurons using RNAscope (Figure 1(A1–A6)). Of 1658 submucosal neurons assessed in 14 WT mice, 1555 (94%) expressed *Nlgn3* mRNA (Figure 1(A7)). In addition to *Nlgn3* mRNA localized to the cell soma, *Nlgn3 mRNA* expression was observed in neuronal and glial fibres (Supplementary Figure S2).

To assess for differential distribution patterns of *Nlgn3* mRNA within the submucosal plexus, the expression profile of *Nlgn3* mRNA in cholinergic neurons (labelled using ChAT immunofluorescence) was determined in 298 neurons from seven WT mice. Almost all cholinergic neurons express *Nlgn3* mRNA (273 of 298 neurons; 92%; Figure 1(B1–B8)). The distribution profile of *Nlgn3* mRNA expression in cholinergic neurons was similar to *Nlgn3* mRNA expression in all submucosal neurons (i.e., labelled by the pan-neuronal marker, Hu; *p* = 0.09; Figure 1(B9)). When we assessed *Nlgn3* mRNA copy number per cell, we found that *Nlgn3* mRNA-expressing cholinergic submucosal neurons contain 30.1 ± 3.8 copies of *Nlgn3* mRNA and that submucosal neurons contain 29.0 ± 1.8 copies (*n* = 298 ChAT and *n* = 1014 neurons; *p* = 0.7; t = 0.29, df = 1310; Figure 1(B10)). 

Next, we measured the expression of *Nlgn3* mRNA in 411 non-cholinergic (VIP-containing) submucosal neurons from seven WT mice (Figure 1(C1–C8)). Nearly all (400 of 411, 97%) VIP-positive neurons express *Nlgn3* mRNA. The distribution profile of *Nlgn3* mRNA expression levels in VIP neurons, however, is skewed to the right and is significantly different from the distribution of *Nlgn3* mRNA in submucosal neurons (*p* < 0.0001) (Figure 1(C9)). Neurons that co-express *Nlgn3* mRNA and VIP contain 28.5 ± 1.3 *Nlgn3* mRNA copies per neuron (*n* = 411 neurons), which is similar to the number of *Nlgn3* mRNA copies expressed in all submucosal neurons sampled from the same preparations overall (25.4 ± 1.0 copies per neuron, *n* = 644 neurons; *p* = 0.06, t = 1.8, df = 1053) (Figure 1(C10)).

### 3.2. Myenteric Neurons Express Nlgn3 mRNA 

**Figure 2:**
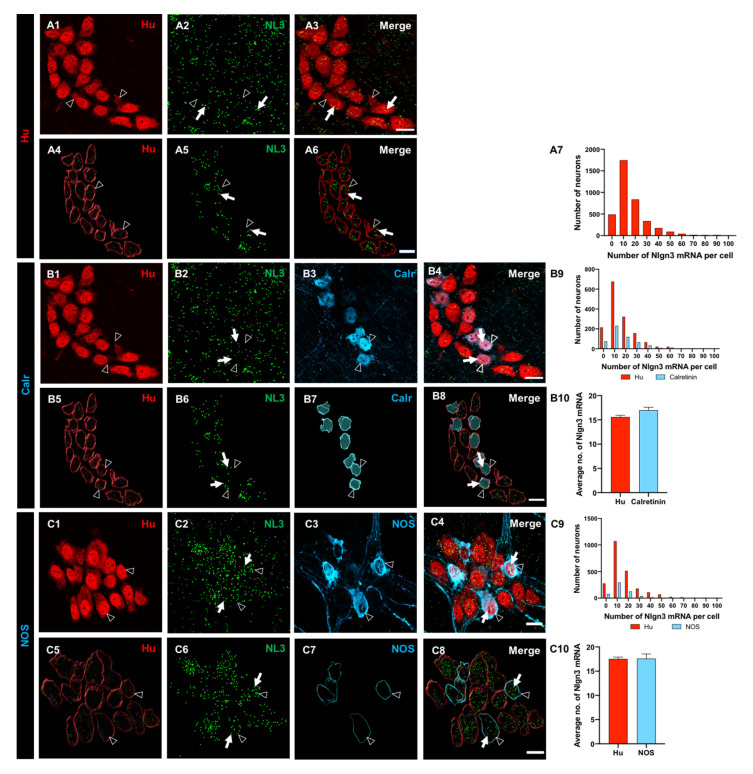
Expression and the distribution of *Nlgn3* mRNA in distal ileal myenteric neurons. (**A1**) Myenteric neurons labelled using Hu immunofluorescence; (**A2**) *Nlgn3* mRNA expression (RNAScope); (**A3**) merge; 3D structure of (**A4**) myenteric neurons, (**A5**) *Nlgn3* mRNA, (**A6**) merge; (**A7**) frequency distribution of *Nlgn3* expression in myenteric neurons. Confocal micrographs of (**B1**) myenteric neurons labelled with the pan-neuronal marker, Hu, (**B2**) *Nlgn3* mRNA, (**B3**) calretinin. (**B4**) Expression of *Nlgn3* mRNA in calretinin neurons; 3D rendering of (**B5**) myenteric neurons, (**B6**) *Nlgn3* mRNA, (**B7**) calretinin, (**B8**) merge; (**B9**) frequency distribution of *Nlgn3* mRNA expression in calretinin-positive myenteric neurons. (**B10**) Calretinin-expressing neurons contain similar copy numbers of *Nlgn3* mRNA to that of myenteric neurons overall. Triple labelling of (**C1**) myenteric neurons, (**C2**) *Nlgn3* mRNA and (**C3**) nNOS expressing myenteric neurons; (**C4**) expression of *Nlgn3* mRNA in nNOS containing myenteric neurons; 3D structure of (**C5**) myenteric neurons, (**C6**) *Nlgn3* mRNA, (**C7**) nNOS-expressing myenteric neurons, (**C8**) *merge*; (**C9**) frequency distribution of *Nlgn3* mRNA in nNOS containing neurons; (**C10**) average number of *Nlgn3* mRNA copies in myenteric nNOS-containing compared to total myenteric neurons. Open arrowheads: Hu staining; arrows: *Nlgn3* mRNA labelling. Scale bar = 10 µm.

The subcellular distribution of *Nlgn3* mRNA in the distal ileum was analysed in 3788 myenteric neurons from 14 WT mice. Most myenteric neurons (labelled with Hu) (3297 of 3788, 87%) expressed *Nlgn3* mRNA within the cell soma. (Figure 2(A1–A6)). Overall, myenteric neurons expressing *Nlgn3* mRNA contained 16.1 ± 0.2 copies of *Nlgn3* mRNA in the cell soma (Figure 2(A7)).

To determine whether *Nlgn3* mRNA is differentially expressed in myenteric neuronal subtypes, we labelled subpopulations using immunofluorescence for cell type-specific markers. Expression data showed that 475 of 550 (86%) of calretinin-immunoreactive neurons express *Nlgn3* mRNA. Frequency distribution analysis of *Nlgn3* mRNA expression levels in calretinin neurons showed a similar profile to the total myenteric neuronal population (total neurons; *n* = 1484, calretinin-positive neurons; *n* = 550; *p* = 0.2). *Nlgn3* mRNA copy numbers in calretinin neurons did not differ significantly from myenteric neuron populations (calretinin-positive neurons; 17.0 ± 0.6 mRNA copies, total neurons; 15.6 ± 0.3 mRNA copies; *p* = 0.3; t = 2.1, df = 2032; Figure 2(B1–B10)).

Co-labelling of nNOS-expressing myenteric neurons with RNAScope revealed that 514 of 593 (87%) nNOS neurons express *Nlgn3* mRNA in the cell soma. The frequency distribution of *Nlgn3* mRNA levels in nNOS-containing neurons was similar to that of total neurons in the myenteric plexus (nNOS positive neurons; *n* = 593, total neurons; *n* = 2304, D = 0.05; *p* = 0.09). Individual nNOS-containing neurons contained an average of 17.6 ± 0.9 *Nlgn3* mRNA copies (*n* = 593 neurons), which was similar to the number of copies expressed in total myenteric neurons (17.5 ± 0.4 *Nlgn3* mRNA copies, *n* = 2304 neurons; *p* = 0.9; t = 0.07, df = 2895; Figure 2(C1–C10)) and in calretinin-positive neurons, as detailed above.

### 3.3. Enteric Glia Express Nlgn3 

**Figure 3:**
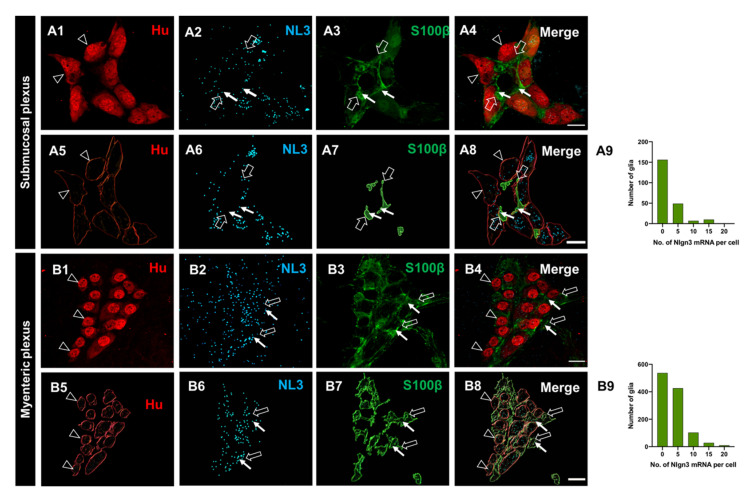
The expression and distribution of *Nlgn3* mRNA in enteric glia. Confocal micrographs of (**A1**) submucosal neurons labelled with the pan-neuronal marker Hu; (**A2**) *Nlgn3* mRNA expression in the submucosal plexus; (**A3**) submucosal glia labelled with S100β; (**A4**) *Nlgn3* mRNA in submucosal glia; Imaris-based 3D rendering of (**A5**) submucosal neurons; (**A6**) *Nlgn3* mRNA, (**A7**) submucosal glia, (**A8**) merge; (**A9**) frequency distribution analysis demonstrates that *Nlgn3* mRNA is expressed in the majority of submucosal glial cells; triple labelling of (**B1**) myenteric neurons using the pan-neuronal marker, Hu, (**B2**) *Nlgn3* mRNA, (**B3**) myenteric glia (using S100β); (**B4**) expression of *Nlgn3* mRNA in myenteric glia; 3D rendering of (**B5**) myenteric neurons, (**B6**) *Nlgn3* mRNA, (**B7**) myenteric glia, (**B8**) merge; (**B9**) frequency distribution analysis indicating that the vast majority of myenteric glia do not express *Nlgn3* mRNA. Open arrowheads: Hu staining, filled arrows: *Nlgn3* mRNA, open arrows: submucosal glia. Scale bar = 10 µm.

Recent studies revealed that *Nlgn3* is also expressed in non-neuronal (enteroendocrine) cells in the intestine [15] but whether *Nlgn3* is expressed in enteric glia has not been assessed. We therefore labelled submucosal glia using an antiserum against the calcium-binding protein, S100β, and assessed for the co-presence of *Nlgn3* mRNA (Figure 3(A1–A4)). Overall, we found that 31% (70 of a total of 226 cells in 6 WT mice) of submucosal glial cells in the mouse ileum express *Nlgn3* mRNA (Figure 3(A1–A9)). Similarly, *Nlgn3* mRNA expression in ileal myenteric glial cells (1203 glial cells in 4 WT mice) was assessed (Figure 3(B1–B8)). In the myenteric plexus, 55% of glial cells (666 of 1203 cells) expressed *Nlgn3* mRNA (Figure 3(B9)). 

### 3.4. ScRNASeq Confirmation of Nlgn3 Expression in Enteric Neurons and Glia

**Figure 4:**
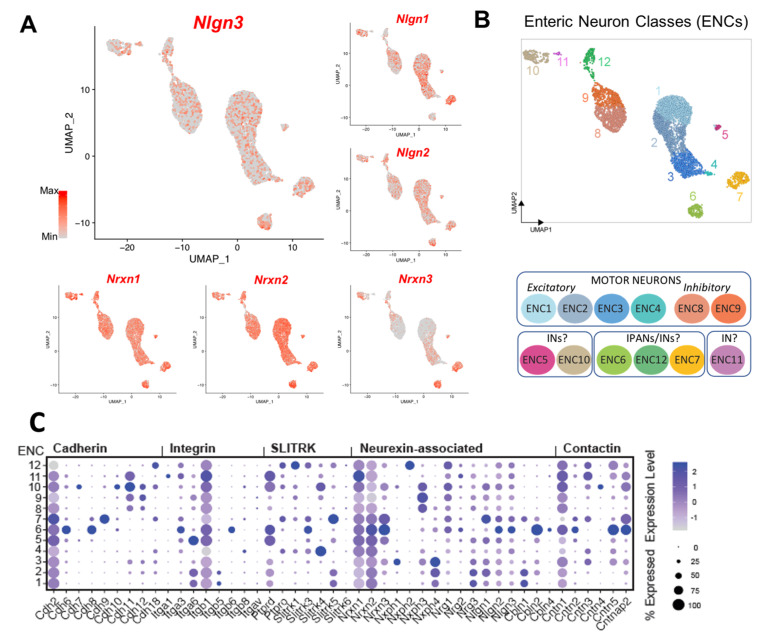
Single cell RNA Sequencing data analysis reveal broad *Nlgn3* expression in enteric neurons. (**A**) UMAP of myenteric neuronal subtypes indicating expression of *Nlgn3* in addition to *Nlgn1* and *Nlgn2* at varying expression levels. Colour bar indicates relative expression level with maximum cut-off at the 90th percentile. (**B**) ENC1-12 displayed on UMAP and schematic indicating plausible functional annotations (modified from [17,18,19,20,21,22,23,24]). (**C**) Dotblots indicating relative expression of *Nrxn* and *Nlgn* genes across ENCs (modified version of figure in [17,18,19,20,21,22,23,24]) UMAP: uniform manifold approximation and projection, IN: Interneuron; IPANS: intrinsic primary afferent neurons. ENC: enteric neuron class.

Our dual RNAScope/immunocytochemistry findings show that most enteric neurons express *Nlgn3* mRNA in mice. To confirm this finding, we investigated both the distribution and levels of *Nlgn3* expression in a published scRNASeq dataset describing molecular cellular subtypes in the juvenile mouse small intestine [16]. In line with our observations using dual RNAScope/immunohistochemistry, scRNASeq data analysis shows *Nlgn3* expression in multiple enteric neuron classes (ENC), including putative intrinsic primary afferent neurons, excitatory and inhibitory motor neurons and interneurons. Given that NLGN3 is thought to form heterodimers, predominantly with NLGN1 in the brain [23], expression patterns for *Nlgn1* and *Nlgn2* in the scRNASeq atlas were also analysed. Unlike in the brain, *Nlgn2* and *Nlgn3* have largely overlapping expression profiles in the enteric nervous system (Figure 4A). *Nlgn1* is expressed in most enteric neuron types, but this expression is less prominent in the ENC4, 11, 12 cell classes. Analysis of the scRNASeq atlas showed that although *Nlgn3* is detected across all neuronal clusters, *Nlgn3* expression levels are highest in ENC6 cells (Figure 4B). Similarly, *Nlgn3* is expressed in the highest proportion of cells in ENC6 (approximately 50% of ENC6 cells express *Nlgn3*) compared to other ENCs (Figure 4C). Based on morphological attributions and projection patterns, ENC6 was validated to correspond to IPANs (intrinsic sensory neurons) of the myenteric plexus [16]. These findings validate our dual RNAScope/immunohistochemistry results to confirm wide-spread expression of *Nlgn3* mRNA in the enteric nervous system and suggest that *Nlgn3* is highly expressed in some IPANs. 

The expression patterns of neuroligin binding partners provide insight into their function in the enteric nervous system. Neurexins are major binding partners of the neuroligin gene family in the brain [23]. Within the enteric nervous system, *Nrxn1* and *Nrxn2* are almost ubiquitously expressed across all ENCs, including robust expression in ENC6 (IPANs). Specifically, *Nrxn1* expression levels are highest in ENC11 (interneurons; INs) whereas *Nrxn2* expression is highest in ENC6 (IPANs), closely followed by strong expression levels in ENCs 1,2 and 4 (putative motor neurons; MNs), 5 (INs) and 7 (IPANs/INs). *Nrxn3* expression levels are highest in ENC 6, 7 (IPANs/INs) and 10 (INs) but are less prominent in other enteric neuronal subtypes (Figure 4C). As lower affinity binding partners of neuroligins, MAM domain-containing glycosylphosphatidylinositol anchor genes (*Mgda1* and *Mdga2*) are expressed at varying levels in myenteric neuron populations (Supplementary Figure S3). 

**Figure 5:**
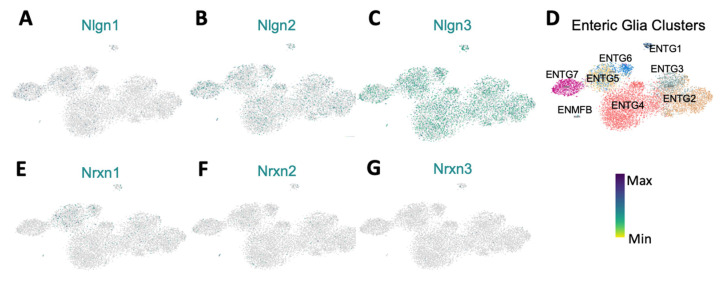
Scatterplots showing differential expression of Neuroligins and Neurexins in enteric glia. (**A**) *Nlgn1* is negligible expressed in EGCs. (**B**) *Nlgn2* is expressed in a low proportion of all ENTGs. (**C**) *Nlgn3* is strongly expressed compared to *Nlgn1* and *Nlgn2* across all ENTGs. (**D**) Representation of enteric glia clusters. (**E**–**G**) *Nrxn1*, *2* and *3* are minimally expressed in enteric glia. Scatterplots were created in Loom viewer from mousebrain.org using scRNA-seq published in Zeisel et al., 2018. Colour bar indicates relative gene expression. ENTG: enteric glia cluster; ENMFB: enteric mesothelial fibroblasts. Pseudocoloured scale bar refers to maximum and minimum expression levels within ENTGs and ENMFB.

The distribution and expression of *Nlgn3* alongside *Nlgn1* and *-2* was also examined in myenteric glia based on scRNASeq data [22]. *Nlgn1* was sparsely expressed in myenteric glial cells (Figure 5A). Although *Nlgn2* was present across all myenteric glia (in a greater proportion of cells than *Nlgn1)*, fewer than 50% of cells assayed contained *Nlgn2* (Figure 5B). In stark contrast, *Nlgn3* was strongly expressed across each myenteric glial cluster (Figure 5C,D). However, of the 10,535 total myenteric glial cells counted, only 4054 cells (38%) express *Nlgn3* mRNA according to the scRNASeq analyses compared to 65% of glial cells as detected using RNAScope. These differences may be due to the lower sensitivity of the scRNASeq approach utilized [22] compared to RNAScope. None of the *Nrxns* (Figure 5E–G) nor *Mgda1* or *Mdga2* (Supplementary Figure S4) were notably expressed in myenteric glia.

### 3.5. The R451C Mutation Selectively Reduces Nlgn3 mRNA Expression 

The R451C mutation reduces Nlgn3 mRNA expression in cholinergic submucosal neurons but not in VIPergic neurons. 

**Figure 6:**
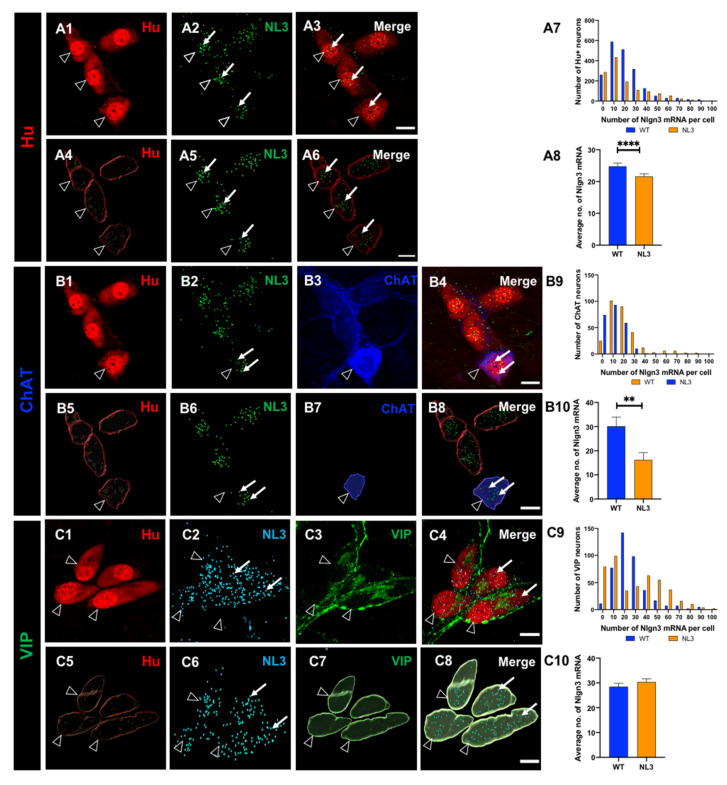
Expression of *Nlgn3* mRNA in the ileal submucosal plexus of *Nlgn3*^R451C^ mutant mice. Confocal micrographs of submucosal neurons labelled with the pan-neuronal marker Hu (**A1**), *Nlgn3* mRNA expression (**A2**); merge (**A3**); the same submucosal neurons shown following 3D reconstruction (**A4**), corresponding cellular *Nlgn3* mRNA expression (**A5**), merge (**A6**); (**A7**) distribution of *Nlgn3* mRNA in WT is significantly different compared to *Nlgn3*^R451C^ mutant mice; (**A8**) *Nlgn3*^R451C^ mutant mice express fewer copies of *Nlgn3* mRNA in submucosal neurons compared to WT; submucosal neurons labelled with Hu pan-neuronal marker (**B1**) *Nlgn3* mRNA (**B2**) and the cholinergic marker, ChAT (**B3**), merge (**B4**); 3D rendering of (**B5**) Hu neurons (**B6**) and corresponding *Nlgn3* mRNA expression (**B7**) and ChAT labelling (**B8**); (**B9**) frequency distribution of *Nlgn3* mRNA in cholinergic submucosal neurons; (**B10**) cholinergic neurons express significantly fewer copies of *Nlgn3* mRNA in *Nlgn3*^R451C^ mutants compared to WT; confocal micrographs of submucosal neurons labelled with (**C1**) the pan-neuronal marker, Hu; (**C2**) *Nlgn3* mRNA labelled using RNAScope and (**C3**) non-cholinergic neurons labelled with VIP; (**C4**) merge; 3D reconstruction of (**C5**) submucosal neurons, (**C6**) corresponding *Nlgn3* mRNA puncta, (**C7**) VIPergic neurons, (**C8**) merge; (**C9**) frequency distribution of *Nlgn3* mRNA in non-cholinergic submucosal neurons; (**C10**) *Nlgn3*^R451C^ mice express similar numbers of *Nlgn3* mRNA copies in non-cholinergic neurons compared to WT in the submucosal plexus. *Nlgn3* mRNA is indicated by the filled arrow, and submucosal glia are labelled with open arrows ** *p* < 0.01, **** *p* < 0.0001, Scale bar = 10 µm.

We next investigated *Nlgn3* mRNA expression in wild-type and *Nlgn3*^R451C^ mutant mouse ileum using dual RNAScope and immunocytochemistry plus Imaris-based quantification (Figure 6(A1–A6)). Frequency distribution analysis of *Nlgn3* mRNA expression in the submucosal neurons labelled with Hu showed that *Nlgn3* mRNA expression is significantly different and that the distribution was skewed to the left in *Nlgn3*^R451C^ mutant mice compared to WT (1985 neurons in 14 WT and 1300 neurons in 10 *Nlgn3*^R451C^ mice were analysed; *p* < 0.0001; Figure 6(A7)). In *Nlgn3*^R451C^ mutant mice, submucosal neuronal cell bodies (*n* = 1300 neurons) express 21.6 ± 0.8 copies of *Nlgn3* mRNA, indicating that significantly less *Nlgn3* mRNA is present in this neuronal subset in mutants than in WT mice (24.8 ± 1.0 copies, *n* = 1985 neurons), *p* = 0.02; t = 2.2, df = 3283 Figure 6(A8)). 

We then characterised the expression of *Nlgn3* mRNA in different submucosal neuronal populations (Figure 6(B1–B8)). Of a total of 241 ChAT neurons in seven *Nlgn3*^R451C^ mice, only 69% (167 neurons) of neurons expressed *Nlgn3* mRNA. The frequency distribution analysis revealed that the distribution of *Nlgn3* mRNA in ChAT neurons in mutant mice is skewed to the left compared to WT (*n* = 298 neurons in 5 WT mice, *n* = 241 neurons in 7 *Nlgn3*^R451C^ mice; D = 0.32; *p* < 0.0001; Figure 6(B9)). Specifically, in *Nlgn3*^R451C^ mutant mice, *Nlgn3* mRNA expression is significantly reduced in ChAT neurons compared to WT (WT; 30.1 ± 3.8 copies, *n* = 298, *Nlgn3*^R451C^ mutant mice; 16.2 ± 2.9 copies, *n* = 241, *p* = 0.006; t = 2.7, df = 537; Figure 6(B10)). 

The impact of the R451C mutation on *Nlgn3* mRNA production in VIP-expressing neurons was also evaluated (Figure 6(C1–C8)). The frequency distribution of the *Nlgn3* mRNA copy numbers in VIP neurons in ileal tissue from *Nlgn3*^R451C^ mice (*n* = 623 neurons in seven WT mice were analysed as well as *n* = 449 neurons in 5 *Nlgn3*^R451C^ mice) is skewed to the left and differs significantly from WT (*p* < 0.0001; Figure 6(C9)). In mutant mice, only 82% (370 of 449) of VIP-containing submucosal neurons express *Nlgn3* mRNA in the cell soma. Of those VIP-expressing neurons, *Nlgn3* mRNA expression levels showed a trend to increase in mutant mice compared to WT (27.1 ± 1.1 versus 30.4 ± 1.2 mRNA copies per cell; *n* = 623 and 449 cells; WT and mutant mice respectively; *p* = 0.05; t = 1.9, df = 1070 Figure 6(C10)).

#### 3.5.1. The R451C Mutation Decreases Nlgn3 mRNA Expression in Calretinin and nNOS-Labelled Myenteric Neurons 

**Figure 7:**
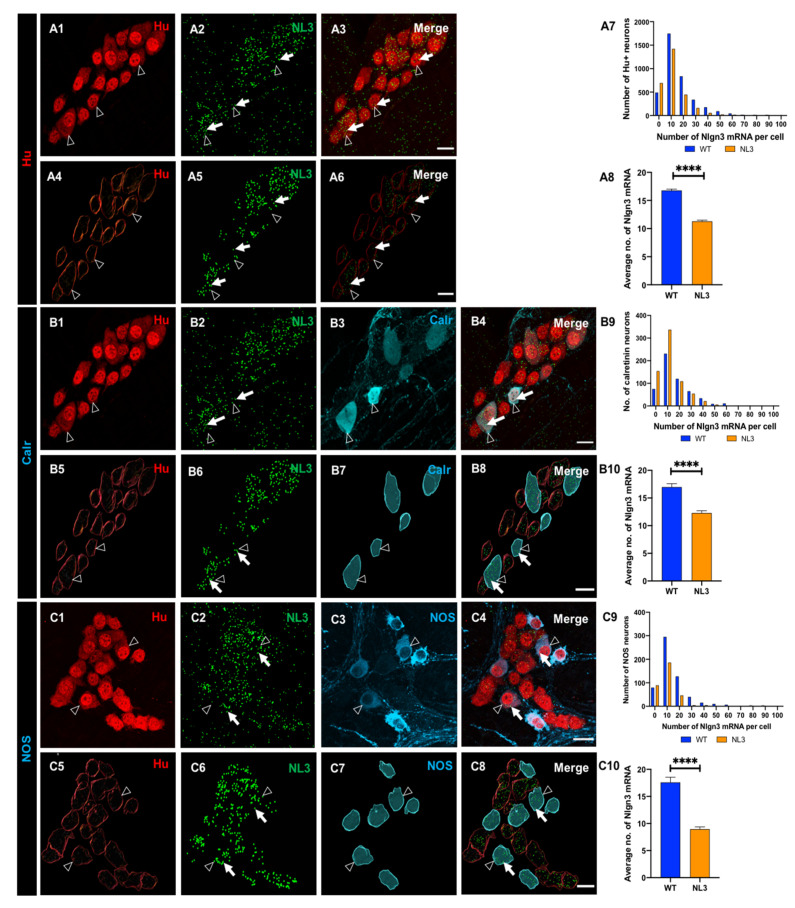
Effects of the *Nlgn3* R451C mutation on *Nlgn3* mRNA expression in myenteric neurons. Confocal images of (**A1**) myenteric neurons (**A2**) *Nlgn3* mRNA expression (**A3**) *Nlgn3* mRNA expression in myenteric neurons; 3D structure of (**A4**) myenteric neurons, (**A5**) *Nlgn3* mRNA, (**A6**) *Nlgn3* mRNA expression in myenteric neurons (**A7**); frequency distribution of *Nlgn3* mRNA in *Nlgn3*^R451C^ mutant mice compared WT (**A8**); in the *Nlgn3*^R451C^ mouse ileum, myenteric neurons express fewer copies of *Nlgn3* mRNA compared to WT; triple labelling of (**B1**) myenteric neurons using (**B2**) the pan-neuronal marker Hu, (**B3**) *Nlgn3* mRNA, (**B3**) calretinin, (**B4**) merge; 3D reconstruction of (**B5**) myenteric neurons, (**B6**) *Nlgn3* mRNA, (**B7**) calretinin expressing neurons and (**B8**) merge; (**B9**) frequency distribution of *Nlgn3* mRNA in calretinin positive myenteric neurons in *Nlgn3*^R451C^ mutant mice and WT; (**B10**) *Nlgn3* R451C mutation reduces *Nlgn3* mRNA expression in mutant mice compared to WT; triple labelling of (**C1**) myenteric neurons, (**C2**) *Nlgn3* mRNA, (**C3**) nNOS expressing neurons, (**C4**) merge; 3D rendering of (**C5**) myenteric neurons, (**C6**) nNOS positive neurons, (**C7**) *Nlgn3* mRNA and (**C8**) merge; (**C9**) frequency distribution of *Nlgn3* mRNA expression in NOS-positive neurons in *Nlgn3*^R451C^ mutant mice; (**C10**) in *Nlgn3*^R451C^ mutant mice, NOS neurons contain fewer *Nlgn3* mRNA copies compared to WT. Filled arrows: *Nlgn3* mRNA. Open arrows: submucosal glia. **** *p* < 0.0001, Scale bar = 10 µm.

We evaluated the impact of the R451C mutation on *Nlgn3* mRNA expression distribution profiles and mRNA copy number in the ileal myenteric plexus (Figure 7(A1–A6)). Using the same approach, we compared *Nlgn3* mRNA expression in total neurons as well as calretinin and nNOS-immunoreactive myenteric neurons in WT and *Nlgn3*^R451C^ mutant mice. We found that the frequency distribution of *Nlgn3* mRNA expression in the myenteric plexus was significantly different (skewed to the left) between WT and *Nlgn3*^R451C^ mice (*p* < 0.0001; Figure 7(A7)). Similar to the findings in the submucosal plexus, *Nlgn3* mRNA copy numbers in myenteric neurons were significantly reduced in *Nlgn3*^R451C^ mutant mice compared to WT (WT; 16.8 ± 0.3 mRNA copies per cell, *Nlgn3*^R451C^ mice; 11.3 ± 0.2 mRNA copies per cell; *n* = 3788, *n* = 2825, WT and *Nlgn3*^R451C^ mutant mice respectively; *p* < 0.0001; t = 15.7, df = 6611; Figure 7(A8)).

We also assessed for differences in *Nlgn3* mRNA expression in calretinin-immunoreactive myenteric neurons (Figure 7(B1–B8)). The frequency distribution of *Nlgn3* mRNA expression in calretinin neurons in *Nlgn3*^R451C^ mice is significantly different (skewed to the left) from the WT (*p* < 0.0001, Figure 7(B9)). In WT, 86% (473 of 550) of calretinin neurons express *Nlgn3* mRNA, whereas in *Nlgn3*^R451C^ mutant mice, 78% of these neurons co-express *Nlgn3* mRNA. Significantly fewer *Nlgn3* mRNA copies were detected in calretinin-immunoreactive neurons in *Nlgn3*^R451C^ mutant mice compared to WT (WT; 17.0 ± 0.6 mRNA copies, *n* = 550 neurons, *Nlgn3*^R451C^; 12.3 ± 0.42 mRNA copies, *n* = 685 neurons; *p* < 0.0001; t = 6.6, df = 1233; Figure 7(B10)).

We previously reported an increased proportion of nNOS neurons in the myenteric plexus of the jejunum and caecum in *Nlgn3*^R451C^ mice [7,24], suggesting that NLGN3 might play an important role in NOS signalling in the myenteric plexus. We therefore analysed the impact of the R451C mutation on *Nlgn3* mRNA expression in nNOS-immunoreactive myenteric neurons (Figure 7(C1–C8)). The frequency distribution of *Nlgn3* mRNA distribution in nNOS immunoreactive neurons in *Nlgn3*^R451C^ mice differs significantly from WT, with this distribution being skewed to the left (*p* < 0.0001, Figure 7(C9)). In WT, about 87% of nNOS-expressing neurons (i.e., 515 of 593 neurons) express *Nlgn3*. Of a total of 331 nNOS-immunoreactive neurons assessed in *Nlgn3*^R451C^ mutant mice, about 73% (241 neurons) co-express *Nlgn3* mRNA. *Nlgn3* mRNA copy numbers were significantly reduced in myenteric nNOS neurons in mutant mice compared to WT (WT; 17.6 ± 0.9 mRNA copies per cell, *n* = 593 neurons, *Nlgn3*^R451C^ mutant mice; 9.0 ± 0.4 mRNA copies per cell, *n* = 331 neurons, *p* < 0.0001, t = 6.6, df = 922; Figure 7(C10)). 

#### 3.5.2. Reduced *Nlgn3* mRNA Expression in Myenteric but Not Submucosal Glia in *Nlgn3^R451C^* Mice 

**Figure 8:**
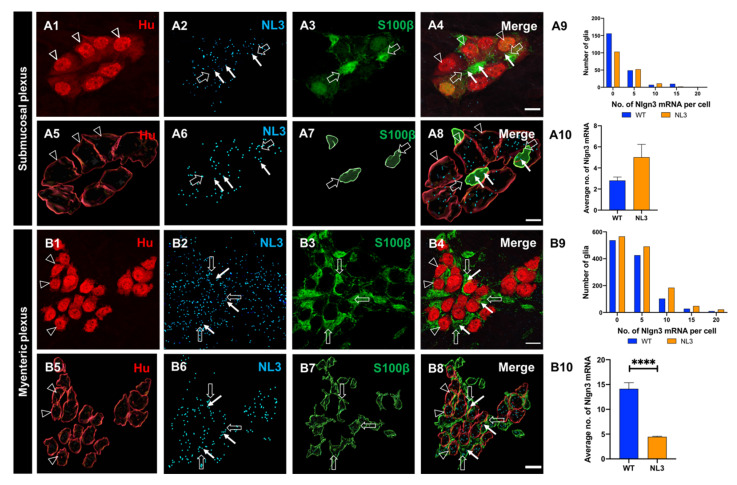
Effects of *Nlgn3* R451C mutation on *Nlgn3* mRNA expression in enteric glia. Confocal images of (**A1**) submucosal neurons labelled with (**A2**) Hu, (**A3**) *Nlgn3* mRNA, (**A4**) merge; 3D structure of (**A5**) submucosal neurons, (**A6**) *Nlgn3* mRNA, (**A7**) submucosal glia, (**A8**) merge; (**A9**) frequency distribution of *Nlgn3* mRNA in submucosal glia in *Nlgn3*^R451C^ mutant mice compared to WT; (**A10**) *Nlgn3* mRNA copy numbers per cell in WT compared to *Nlgn3*^R451C^ mutant mice; immunofluorescent labelling of (**B1**) myenteric neurons (Hu), (**B2**) *Nlgn3* mRNA, (**B3**) myenteric glia (S100β), (**B4**) merge; 3D reconstruction of (**B5**) myenteric neurons, (**B6**) *Nlgn3* mRNA, (**B7**) myenteric glia, (**B8**) merge; (**B9**) frequency distribution of *Nlgn3* mRNA expression in WT and *Nlgn3*^R451C^ mutant mice. (**B10**) Mutant mice express significantly fewer *Nlgn3* mRNA copies in myenteric glia compared to WT. **** *p* < 0.0001, Scale bar = 10 µm.

As described earlier, *Nlgn3* mRNA is expressed in both submucosal and myenteric glia in the WT mouse ileum. *Nlgn3* mRNA expression was assessed in of the ileal submucosal plexus in WT (*n* = 6) and *Nlgn3*^R451C^ (*n* = 5) mutant mice, respectively (Figure 8(A1–A8)). In *Nlgn3*^R451C^ mutant mice, 42% (73 of a total of 173) of submucosal glial cells expressed *Nlgn3* mRNA. The frequency distribution data of *Nlgn3* mRNA expression in submucosal glial cells show similar levels of *Nlgn3* mRNA in WT and *Nlgn3*^R451C^ (*p* = 0.09, Figure 8(A9)). In submucosal glia, there was a trend for an increase in mRNA copy number in *Nlgn3*^R451C^ mutants (2.8 ± 0.3 mRNA copies and 5.0 ± 1.2 mRNA copies in WT and *Nlgn3*^R451C^ mice, respectively, *p* = 0.05; t = 1.9, df = 397, Figure 8(A10)).

We also compared *Nlgn3* mRNA expression in myenteric glia in WT and *Nlgn3*^R451C^ mutant mice. In *Nlgn3*^R451C^ mutant mice, only 37% (496 of a total of 1318) of myenteric glial cells expressed *Nlgn3* mRNA (Figure 8(B1–B8)), and the frequency distribution of *Nlgn3* mRNA in myenteric glia in mutant mice was significantly different (skewed to the left) to WT (*n* = 1203 glial cells in 5 WT mice, *n* = 1318 cells in 5 *Nlgn3*^R451C^ mice; *p* = 0.001, Figure 8(B9)). *Nlgn3* mRNA expression was reduced in myenteric glia in *Nlgn3*^R451C^ mice compared to WT (WT; 14.2 ± 1.2 mRNA copies/cell, *n* = 1203 cells, *Nlgn3*^R451C^; 4.4 ± 0.1 mRNA copies/cell, *n* = 1318 cells, *p* < 0.0001, t = 8.2, df = 2519, Figure 8(B10)).

Overall, we found that *Nlgn3* mRNA is expressed in the mouse enteric nervous system (including enteric glia) and that *Nlgn3^R451C^* mice have reduced *Nlgn3* mRNA expression in subpopulations of submucosal and myenteric plexus neurons (Table 3). The molecular identities of enteric neuronal populations have now been well described in mice based on scRNASeq combined with immunostaining verification [16,22]. These studies show the presence of 12 enteric neuronal populations with distinct neurochemical signatures and enteric glia. We therefore investigated the cellular expression of *Nlgn3* in the mouse enteric nervous system based on previously reported scRNASeq datasets and found that all enteric neuronal subtypes and glia express *Nlgn3* at varying levels. In mice, the R451C mutation reduces *Nlgn3* mRNA expression in submucosal cholinergic as well as myenteric nitrergic and calretinin neurons. *Nlgn3^R451C^* mice also had reduced mRNA expression levels in myenteric (but not submucosal) glia. In contrast, *Nlgn3* mRNA expression was unchanged in VIPergic submucosal neurons in *Nlgn3^R451C^* mice. Taken together, these findings suggest that NLGN3 is an important component of neuronal and neuron–glial communication in the gut and that mutations in NLGN3 might play a role in GI dysfunction in individuals with autism. 

## 4. Discussion

In this study, we showed that most submucosal and myenteric neurons in the mouse ileum express *Nlgn3* mRNA. For the first time, we revealed that *Nlgn3* mRNA is expressed in ileal submucosal and myenteric glia. In addition, we report that the autism-associated *Nlgn3* R451C mutation reduces *Nlgn3* mRNA expression in both submucosal and myenteric neurons as well as myenteric glia in the mouse ileum. 

### 4.1. Most Enteric Neurons Express Nlgn3 mRNA 

Although there is ample evidence for the existence of prominent postsynaptic densities within the ENS [25,26,27,28], the molecular characterization remains to be fully characterized. In the rodent brain, neuroligin-3 is expressed at both excitatory and inhibitory synapses [8] and is closely associated with other PDZ-associated molecules to mediate postsynaptic signal transduction. There is evidence that PDZ domain proteins such as postsynaptic density protein 95 (PSD95) [29,30], postsynaptic density protein 93 (PSD93) [30], as well as the cell adhesion molecules L1 [31] and neuroligin-1 [32] are expressed in the ENS. 

#### 4.1.1. Cholinergic Transmission

It is well established that NLGN3 is involved in both excitatory and inhibitory synaptic transmission in the brain. However, relatively little is known about the distribution of postsynaptic proteins associated with excitatory (predominantly mediated via nicotinic acetylcholine receptors (nAChRs)) and inhibitory synapses in the ENS. We reveal that most cholinergic submucosal neurons also contain *Nlgn3* mRNA. Within the enteric neural circuitry, acetylcholine is the primary excitatory neurotransmitter utilised by cholinergic secretomotor neurons, excitatory muscle motor neurons, ascending interneurons, descending interneurons and intrinsic sensory neurons [33,34,35,36]. Since virtually all submucosal neurons receive synaptic inputs via cholinergic synapses [18], *Nlgn3* is likely present in cholinergic synapses in the submucosal plexus. All submucosal neurons receive cholinergic fast excitatory postsynaptic potentials and hence can be expected to have postsynaptic densities with clusters of nicotinic receptors and NLGNs as a part of the synaptic structure. Although there is no available evidence of NLGN3 expression in the cholinergic system of the central or peripheral nervous systems, expression of other NLGN subtypes in cholinergic synapses has been reported. For example, NLGN2 is expressed at the postsynaptic membrane of cholinergic synapses in the mouse brain [37], and NLGN1 is present in cholinergic synapses in the chick ciliary ganglion [38]. Since NLGN3 and other NLGN isoforms can be colocalized in the same synapse [8], NLGN3 might be co-expressed with NLGN1 and NLGN2 in cholinergic synapses in the submucosal plexus. 

#### 4.1.2. VIP-Expressing Neurons

We also revealed that *Nlgn3* mRNA is expressed in VIP-expressing submucosal neurons in the mouse ileum. In the submucosal plexus, VIP is a primary neurotransmitter of secretomotor neurons and stimulates intestinal secretion [18,39]. In the myenteric plexus, VIP is expressed in inhibitory muscle motor neurons and interneurons [21,40]. VIP also acts as a co-transmitter alongside ACh and NO in a subset of descending interneurons in the mouse and guinea pig ileum [20,21,41]. Moreover, an excitatory role for the vasoactive intestinal peptide 1 (VPAC1) receptor on cholinergic neurons has been identified in the myenteric plexus [42]. These findings open a novel avenue for research in *Nlgn3^R451C^* mice since the expression profiles of NLGN3 or other NLGN isoforms in VIP-containing synapses have not been reported to date in either the CNS or the peripheral nervous system. 

#### 4.1.3. nNOS-Expressing Neurons

One of the major findings of this study is that most NO-containing enteric neurons express *Nlgn3* mRNA in the cell soma. Nitric oxide (NO) is the predominant inhibitory neurotransmitter to the smooth muscle in the enteric nervous system [20,21] and is also expressed in descending interneurons together with other neurotransmitters [43]. Given that NLGN complexes anchor postsynaptic densities and PSD95 has at least one nNOS binding PDZ domain, *Nlgn3* may contribute to synaptic specialization of enteric NO neurons. However, the distributions of PSD95 and PSD93 have not been well characterized in ENS. It has been shown that PSD93 interacts with nNOS via a PDZ-PDZ domain interaction [30]. Some nNOS expressing myenteric neurons express PSD93 [44]. In agreement with this finding, our data indicating *Nlgn3* expression in this neuronal population suggest that Nlgn3 might be expressed at the postsynaptic membrane of nNOS immunoreactive myenteric neurons.

#### 4.1.4. Calretinin-Expressing Neurons 

We also show *Nlgn3* mRNA expression in calretinin-expressing myenteric neurons in the mouse ileum. In the enteric nervous system, calretinin is expressed in intrinsic primary afferent neurons, interneurons and excitatory motor neurons innervating the smooth muscle layer [20,21]. In line with these findings, scRNASeq data analysis confirmed expression of *Nlgn3* at varying levels in mouse myenteric neurons, including putative motor neurons, interneurons and intrinsic primary afferent neurons in the mouse small intestine. It has been reported that the PSD93 protein is localized to calretinin neurons in mouse myenteric plexus [44]. Therefore, it is possible that *Nlgn3* is associated with postsynaptic protein complexes within calretinin neurons in the myenteric plexus.

#### 4.1.5. Other Signalling Systems 

Given that glutamate and GABA have active roles in the ENS [45,46] alongside the presence of postsynaptic proteins including PSD93 [44], the wide distribution of NLGN3 we observed in the ENS suggests that NLGN3 could also be expressed at glutamatergic and GABAergic synapses within the ENS.

### 4.2. Differential Expression of Neuroligin Binding Partners in the Enteric Nervous System

When examining scRNASeq data, *Nlgn2* and *3* expression profiles are almost identical in all classes of enteric neurons. In contrast with findings in the central nervous system [8] reviewed by [47], this suggests that NLGN3 and NLGN2 co-express in enteric neurons subtypes, and we speculate that they may form heterodimers in the enteric nervous system. Expression patterns of the neurexin family of neuroligin binding partners, however, show that *Nrxn2* and *Nrxn3* are more strongly expressed within the enteric neuronal cell clusters compared with *Nrxn1*. These findings suggest that *Nlgn3* may preferentially bind with *Nrxn2* and *3* at synapses in IPANs of the myenteric plexus. In the CNS, NLGNs are present at classical synapses receiving fast excitatory postsynaptic potentials (EPSPs), but the most functional evidence suggests that IPANs do not exhibit fast EPSPs. For example, findings from Hibberd and colleagues [48] indicate that IPANs receive fast EPSPs, but in contrast, both [49,50] reported that AH neurons do not exhibit fast EPSPs. Nevertheless, it is unclear whether junctions producing slow EPSPs might express neuroligin complexes; therefore, further research is required to identify the role of NLGN3 in IPANs in the ENS.

### 4.3. A Role for Nlgn3 in Enteric Neuronal-Glial Synapses

For the first time, we reveal that *Nlgn3* mRNA is expressed in enteric glia. The higher proportion of myenteric glia represents approximately a three-fold enrichment of *Nlgn3* expression compared to submucosal *Nlgn3*-expressing glia and may provide a useful way to chemically identify and assist in identifying the function of glial subtypes in the gut. scRNAseq analysis showed that most enteric glia express *Nlgn3* mRNA but negligible/low levels of *Nlgn1* and *Nlgn2*. Therefore, we propose that NLGN3 acts as a main adhesion protein in glia for modulating glial-neuron synaptic activity in the enteric nervous system.

Enteric glia play a major role in enteric nervous system-mediated GI functions, including mucosal secretion, intestinal permeability, mucosal sensation, GI motility and immune responses [51,52,53] in concert with enteric neurons. Enteric neurons synapse onto enteric glia to regulate enteric nervous system-coordinated GI responses [51,54,55]. Enteric glial–neuronal associations revealed by immunocytochemistry highlight that enteric glia are in close contact with nerve fibres and varicose release sites [56]. Evidence for neuron–glia communication also comes from live imaging experiments reporting the presence of several signalling pathways. Ca^2+^ imaging studies indicate that neuron–glia communication is regulated by neurally released purines which activate purinergic receptors on enteric glia [55,56,57,58]. In the submucosal plexus, neuron–glial transmission occurs via P2Y_1_ and P2Y_4_ receptors [59]. Furthermore, neuronal ATP release via the pannexin-1 channel also influences neuron–glia interactions [60,61]. Based on this evidence, we propose that NLGN3 expressed by both enteric neurons and glia modulate neuron–glia signalling pathways. This hypothesis is well established in the mouse brain where RNA sequencing of the transcriptome revealed that *Nlgn3* transcripts are enriched in glial cell types, including astrocytes and oligodendrocytes [62]. NLGNs expressed in astrocytes aid in neuron–astrocyte communication via bi- and tripartite synapses in mice in *C. elegans* [63], suggesting that a neuronal–glial communication role may not be specific to mice. Similarly, NLGN3 expressed in enteric glia could also be involved in establishing bi-partite and tripartite synapses in the enteric nervous system. Given this evidence, this study strongly suggests that NLGN3 might play a role in synapse formation and modifying synaptic function during neuron–glia communication in the enteric nervous system.

### 4.4. The R451C Mutation Reduces Nlgn3 mRNA Expression in the Enteric Nervous System 

We previously demonstrated that mice expressing the *Nlgn3* R451C mutation have faster small intestinal transit and increased numbers of myenteric neurons in the small intestine [7]. How this mutation affects NLGN3 expression in the enteric nervous system, however, is not understood. Here, we show that the R451C mutation decreases *Nlgn3* mRNA expression in both neurons and glia in the mouse ileal submucosal plexus. For example, cholinergic submucosal neurons in mutant mice contain fewer *Nlgn3* mRNA copies compared to WT. These changes could potentially alter cholinergic signalling in the submucosal plexus, which could affect functions mediated by cholinergic neurons such as secretion, absorption and mucosal barrier functions. Although there are no reports available on changes to the cholinergic system of the enteric nervous system in ASD, CNS studies showed that altered cholinergic neurons are associated with the pathophysiology of autism [64,65]. Specifically, ASD patient basal forebrain tissues show altered cholinergic neuronal numbers, size and structure [66]. In addition, a decreased plasma concentration of choline, a precursor for acetylcholine, has been reported in ASD patients [67,68], and reduced levels of hippocampal cytosolic choline have been correlated with autism severity [67]. In the human GI tract, NLGN3-mediated alterations to the cholinergic system could induce GI dysfunction; however, further studies are required to determine effects of the R451C mutation on cholinergic signalling in the enteric nervous system.

In the myenteric plexus of the distal ileum, the R451C mutation reduces *Nlgn3* mRNA expression levels in neurons. Specifically, *Nlgn3* mRNA expression is substantially reduced in both calretinin and nNOS-immunoreactive neuronal populations. Although the contribution of calretinin neurons to ASD or ASD-related GI pathophysiology is unclear, the significance of calretinin neurons in other ENS-related diseases has been highlighted [69]. As mentioned, an increased proportion of NOS1 expressing neurons has previously been observed in *Nlgn3R451C* mice [7], and our current findings showing reduced *Nlgn3* expression in these cells might contribute to altered NO neuronal signalling in the enteric nervous system in these mice. 

Finally, the impact of the R451C mutation on *Nlgn3* expression in glia has not previously been reported. Here, we show that in *Nlgn3*^R451C^ mice, *Nlgn3* mRNA expression is dramatically reduced in myenteric glia but is unchanged in glia located within the submucosal plexus. Since glia play an important role in mediating intestinal functions such as mucosal barrier regulation, gut motility, immune responses and neurotransmission, such a reduction in NLGN3 levels could contribute to GI dysfunction in *Nlgn3*^R451C^ mutant mice. Therefore, in addition to neuronal dysfunction, glial dysfunction could also contribute to GI pathology in individuals diagnosed with ASD.

## 5. Conclusions

Here, we characterised *Nlgn3* mRNA expression and identified effects of the autism-associated R451C mutation in this gene in the enteric nervous system of the mouse ileum. We show that *Nlgn3* mRNA is expressed in situ in most enteric neuronal and glial populations of the myenteric and submucosal plexuses in the mouse ileum. Our RNAseq analysis confirms the presence of *Nlgn3* in myenteric neuronal subtypes and glia in mice. We found that *Nlgn3*, but not *Nlgn1* or *2,* is expressed at high levels in enteric glia, whereas glial expression of major binding partners for neuroligins, such as neurexins 1–3, is negligible. We therefore propose that neuroligin-3 is an important glial adhesion protein in neuronal–glial synapses in the mouse enteric nervous system. We demonstrate that the R451C mutation differentially reduces *Nlgn3* mRNA expression in the enteric nervous system, particularly in cholinergic submucosal neurons, myenteric calretinin and NOS1-expressing neurons and myenteric glia in the mouse ileum. Taken together, these findings suggest that neuroligin-3 plays a role in regulating enteric nervous system-mediated GI physiology and that changes to *Nlgn3* mRNA expression could contribute to GI dysfunction in the *Nlgn3^R451C^* mouse model of autism. 

## Figures and Tables

**Table 1:** Primary antibodies used for immunocytochemistry.

Primary Antisera	Cell Type	Raised in	Dilution	Source
ANNA-1(anti HuC/D) *	All neurons	Human	1:5000	Gift from Dr. V. Lennon
VIP	Non-cholinergic secretomotor neurons	Rabbit	1:1000	Merck Millipore
ChAT	Cholinergic secretomotor neurons	Goat	1:100	Chemicon
nNOS	Inhibitory muscle motor neurons	Sheep	1:1000	Gift from Dr P. Emson
Calretinin	Interneurons, IPANS, excitatory motor neurons	Goat	1:1000	SWANT
S100β	Enteric glia	Rabbit	1:1000	DAKO

* Referred to as Hu throughout the text.

**Table 2:** Secondary antibodies used for immunocytochemistry.

Secondary Antisera	Raised in	Dilution	Source
Anti-human AF 594	Donkey	1:500	Jackson Immuno Labs
Anti-sheep AF 647	Donkey	1:500	Molecular Probes
Anti-rabbit AF 647	Donkey	1:400	Molecular Probes
Anti-sheep AF 594	Donkey	1:100	Molecular Probes

**Table 3:** Comparison of *Nlgn3* copy number expression in neuronal and glial subtypes in the submucosal and myenteric plexus in *Nlgn3*^R451C^ and WT mice.

Enteric Neuronal Plexus	Cell Type	WT (Mean ± SEM)	*Nlgn3*^R451C^ (Mean ± SEM)	*p* Value
Submucosal plexus	Hu	24.8 ± 1.0	21.6 ± 0.8	0.02
	VIP	27.1 ± 1.1	30.4 ± 1.2	0.05
	ChAT	30.1 ± 3.8	16.2 ± 2.9	0.006
	S100β	2.8 ± 0.3	5.0 ± 1.2	0.05
Myenteric plexus	Hu	16.8 ± 0.3	11.3 ± 0.2	<0.0001
	Calretinin	17.0 ± 0.6	12.3 ± 0.42	<0.0001
	nNOS	17.6 ± 0.9	9.0 ± 0.4	<0.0001
	S100β	14.2 ± 1.2	4.4 ± 0.1	<0.0001

## Data Availability

Data supporting the findings of this study are available from the corresponding author upon request.

## Supplementary Materials

The following supporting information can be downloaded at: https://www.mdpi.com/article/10.3390/biom13071063/s1, Figure S1: Negative control experiments for the myenteric plexus and the submucosal plexus; Figure S2: *Nlgn3* mRNA expression in submucosal neurons, glia and glial fibers; Figure S3: Expression of NLGN3 potential binding partners MDGA1 and 2 in mouse myenteric neurons; Figure S4: Expression of NLGN3 potential binding partners MDGA1 and 2 in mouse enteric glia; Table S1: Number of mice used for identifying enteric nervous system cellular subtypes.

## References

[1] Tabuchi K., Blundell J., Etherton M.R., Hammer R.E., Liu X., Powell C.M., Sudhof T.C. (2007). A neuroligin-3 mutation implicated in autism increases inhibitory synaptic transmission in mice. Science.

[2] Etherton M., Foldy C., Sharma M., Tabuchi K., Liu X., Shamloo M., Malenka R.C., Sudhof T.C. (2011). Autism-linked neuroligin-3 R451C mutation differentially alters hippocampal and cortical synaptic function. Proc. Natl. Acad. Sci. USA.

[3] Foldy C., Malenka R.C., Sudhof T.C. (2013). Autism-associated neuroligin-3 mutations commonly disrupt tonic endocannabinoid signaling. Neuron.

[4] Rothwell P.E., Fuccillo M.V., Maxeiner S., Hayton S.J., Gokce O., Lim B.K., Fowler S.C., Malenka R.C., Sudhof T.C. (2014). Autism-associated neuroligin-3 mutations commonly impair striatal circuits to boost repetitive behaviors. Cell.

[5] Hosie S., Malone D.T., Liu S., Glass M., Adlard P.A., Hannan A.J., Hill-Yardin E.L. (2018). Altered Amygdala Excitation and CB1 Receptor Modulation of Aggressive Behavior in the Neuroligin-3(R451C) Mouse Model of Autism. Front. Cell. Neurosci..

[6] Leembruggen A.J.L., Balasuriya G.K., Zhang J., Schokman S., Swiderski K., Bornstein J.C., Nithianantharajah J., Hill-Yardin E.L. (2019). Colonic dilation and altered ex vivo gastrointestinal motility in the neuroligin-3 knockout mouse. Autism Res..

[7] Hosie S., Ellis M., Swaminathan M., Ramalhosa F., Seger G.O., Balasuriya G.K., Gillberg C., Rastam M., Churilov L., McKeown S.J. (2019). Gastrointestinal dysfunction in patients and mice expressing the autism-associated R451C mutation in neuroligin-3. Autism Res..

[8] Budreck E.C., Scheiffele P. (2007). Neuroligin-3 is a neuronal adhesion protein at GABAergic and glutamatergic synapses. Eur. J. Neurosci..

[9] Venkatesh H.S., Johung T.B., Caretti V., Noll A., Tang Y., Nagaraja S., Gibson E.M., Mount C.W., Polepalli J., Mitra S.S. (2015). Neuronal Activity Promotes Glioma Growth through Neuroligin-3 Secretion. Cell.

[10] Gilbert M., Smith J., Roskams A.J., Auld V.J. (2001). Neuroligin 3 is a vertebrate gliotactin expressed in the olfactory ensheathing glia, a growth-promoting class of macroglia. Glia.

[11] Jamain S., Quach H., Betancur C., Rastam M., Colineaux C., Gillberg I.C., Soderstrom H., Giros B., Leboyer M., Gillberg C. (2003). Mutations of the X-linked genes encoding neuroligins NLGN3 and NLGN4 are associated with autism. Nat. Genet..

[12] Sanders S.J., Ercan-Sencicek A.G., Hus V., Luo R., Murtha M.T., Moreno-De-Luca D., Chu S.H., Moreau M.P., Gupta A.R., Thomson S.A. (2011). Multiple recurrent de novo CNVs, including duplications of the 7q11.23 Williams syndrome region, are strongly associated with autism. Neuron.

[13] Levy D., Ronemus M., Yamrom B., Lee Y.H., Leotta A., Kendall J., Marks S., Lakshmi B., Pai D., Ye K. (2011). Rare de novo and transmitted copy-number variation in autistic spectrum disorders. Neuron.

[14] Zhang Q., Wang J., Li A., Liu H., Zhang W., Cui X., Wang K. (2013). Expression of neurexin and neuroligin in the enteric nervous system and their down-regulated expression levels in Hirschsprung disease. Mol. Biol. Rep..

[15] Bohorquez D.V., Shahid R.A., Erdmann A., Kreger A.M., Wang Y., Calakos N., Wang F., Liddle R.A. (2015). Neuroepithelial circuit formed by innervation of sensory enteroendocrine cells. J. Clin. Investig..

[16] Morarach K., Mikhailova A., Knoflach V., Memic F., Kumar R., Li W., Ernfors P., Marklund U. (2021). Diversification of molecularly defined myenteric neuron classes revealed by single-cell RNA sequencing. Nat. Neurosci..

[17] Wang F., Flanagan J., Su N., Wang L.C., Bui S., Nielson A., Wu X., Vo H.T., Ma X.J., Luo Y. (2012). RNAscope: A novel in situ RNA analysis platform for formalin-fixed, paraffin-embedded tissues. J. Mol. Diagn..

[18] Foong J.P., Tough I.R., Cox H.M., Bornstein J.C. (2014). Properties of cholinergic and non-cholinergic submucosal neurons along the mouse colon. J. Physiol..

[19] Young H.M., Ciampoli D. (1998). Transient expression of neuronal nitric oxide synthase by neurons of the submucous plexus of the mouse small intestine. Cell Tissue Res..

[20] Qu Z.D., Thacker M., Castelucci P., Bagyanszki M., Epstein M.L., Furness J.B. (2008). Immunohistochemical analysis of neuron types in the mouse small intestine. Cell Tissue Res..

[21] Sang Q., Young H.M. (1996). Chemical coding of neurons in the myenteric plexus and external muscle of the small and large intestine of the mouse. Cell Tissue Res..

[22] Zeisel A., Hochgerner H., Lonnerberg P., Johnsson A., Memic F., van der Zwan J., Haring M., Braun E., Borm L.E., La Manno G. (2018). Molecular Architecture of the Mouse Nervous System. Cell.

[23] Sudhof T.C. (2008). Neuroligins and neurexins link synaptic function to cognitive disease. Nature.

[24] Sharna S.S., Balasuriya G.K., Hosie S., Nithianantharajah J., Franks A.E., Hill-Yardin E.L. (2020). Altered Caecal Neuroimmune Interactions in the Neuroligin-3(R451C) Mouse Model of Autism. Front. Cell. Neurosci..

[25] Gabella G. (1972). Fine structure of the myenteric plexus in the guinea-pig ileum. J. Anat..

[26] Llewellyn-Smith I.J., Costa M., Furness J.B. (1985). Light and electron microscopic immunocytochemistry of the same nerves from whole mount preparations. J. Histochem. Cytochem..

[27] Young H.M., Furness J.B. (1995). Ultrastructural examination of the targets of serotonin-immunoreactive descending interneurons in the guinea pig small intestine. J. Comp. Neurol..

[28] Pompolo S., Furness J.B. (1988). Ultrastructure and synaptic relationships of calbindin-reactive, Dogiel type II neurons, in myenteric ganglia of guinea-pig small intestine. J. Neurocytol..

[29] Chaudhury A., He X.D., Goyal R.K. (2009). Role of PSD95 in membrane association and catalytic activity of nNOSalpha in nitrergic varicosities in mice gut. Am. J. Physiol. Gastrointest. Liver Physiol..

[30] Brenman J.E., Christopherson K.S., Craven S.E., McGee A.W., Bredt D.S. (1996). Cloning and characterization of postsynaptic density 93, a nitric oxide synthase interacting protein. J. Neurosci..

[31] Anderson R.B., Turner K.N., Nikonenko A.G., Hemperly J., Schachner M., Young H.M. (2006). The cell adhesion molecule l1 is required for chain migration of neural crest cells in the developing mouse gut. Gastroenterology.

[32] Zheng Y., Lv X., Wang D., Gao N., Zhang Q., Li A. (2017). Down-regulation of fibronectin and the correlated expression of neuroligin in hirschsprung disease. Neurogastroenterol. Motil..

[33] Brookes S.J. (2001). Classes of enteric nerve cells in the guinea-pig small intestine. Anat. Rec..

[34] Grider J.R. (2003). Neurotransmitters mediating the intestinal peristaltic reflex in the mouse. J. Pharmacol. Exp. Ther..

[35] Gwynne R.M., Bornstein J.C. (2007). Synaptic transmission at functionally identified synapses in the enteric nervous system: Roles for both ionotropic and metabotropic receptors. Curr. Neuropharmacol..

[36] Brookes S.J., Steele P.A., Costa M. (1991). Identification and immunohistochemistry of cholinergic and non-cholinergic circular muscle motor neurons in the guinea-pig small intestine. Neuroscience.

[37] Takacs V.T., Freund T.F., Nyiri G. (2013). Neuroligin 2 is expressed in synapses established by cholinergic cells in the mouse brain. PLoS ONE.

[38] Ross B.S., Conroy W.G. (2008). Capabilities of neurexins in the chick ciliary ganglion. Dev. Neurobiol..

[39] Mongardi Fantaguzzi C., Thacker M., Chiocchetti R., Furness J.B. (2009). Identification of neuron types in the submucosal ganglia of the mouse ileum. Cell Tissue Res..

[40] Keef K.D., Shuttleworth C.W., Xue C., Bayguinov O., Publicover N.G., Sanders K.M. (1994). Relationship between nitric oxide and vasoactive intestinal polypeptide in enteric inhibitory neurotransmission. Neuropharmacology.

[41] Costa M., Brookes S.J., Steele P.A., Gibbins I., Burcher E., Kandiah C.J. (1996). Neurochemical classification of myenteric neurons in the guinea-pig ileum. Neuroscience.

[42] Fung C., Unterweger P., Parry L.J., Bornstein J.C., Foong J.P. (2014). VPAC1 receptors regulate intestinal secretion and muscle contractility by activating cholinergic neurons in guinea pig jejunum. Am. J. Physiol. Gastrointest. Liver Physiol..

[43] Young H.M., Furness J.B., Povey J.M. (1995). Analysis of connections between nitric oxide synthase neurons in the myenteric plexus of the guinea-pig small intestine. J. Neurocytol..

[44] Swaminathan M., Foong JP P., Hill-Yardin E.L., Bornstein J.C. (2016). PSD-93 is expressed in most, but not all, myenteric neurons in the mouse colon. Neurogastroenterol. Motil..

[45] Swaminathan M., Hill-Yardin E.L., Bornstein J.C., Foong J.P.P. (2019). Endogenous Glutamate Excites Myenteric Calbindin Neurons by Activating Group I Metabotropic Glutamate Receptors in the Mouse Colon. Front. Neurosci..

[46] Seifi M., Brown J.F., Mills J., Bhandari P., Belelli D., Lambert J.J., Rudolph U., Swinny J.D. (2014). Molecular and functional diversity of GABA-A receptors in the enteric nervous system of the mouse colon. J. Neurosci..

[47] Sudhof T.C. (2017). Synaptic Neurexin Complexes: A Molecular Code for the Logic of Neural Circuits. Cell.

[48] Hibberd T.J., Travis L., Wiklendt L., Costa M., Brookes S.J.H., Hu H., Keating D.J., Spencer N.J. (2018). Synaptic activation of putative sensory neurons by hexamethonium-sensitive nerve pathways in mouse colon. Am. J. Physiol. Gastrointest. Liver Physiol..

[49] Foong J.P., Nguyen T.V., Furness J.B., Bornstein J.C., Young H.M. (2012). Myenteric neurons of the mouse small intestine undergo significant electrophysiological and morphological changes during postnatal development. J. Physiol..

[50] Nurgali K., Stebbing M.J., Furness J.B. (2004). Correlation of electrophysiological and morphological characteristics of enteric neurons in the mouse colon. J. Comp. Neurol..

[51] Gulbransen B.D., Sharkey K.A. (2012). Novel functional roles for enteric glia in the gastrointestinal tract. Nat. Rev. Gastroenterol. Hepatol..

[52] Grubisic V., Verkhratsky A., Zorec R., Parpura V. (2018). Enteric glia regulate gut motility in health and disease. Brain Res. Bull..

[53] Broadhead M.J., Bayguinov P.O., Okamoto T., Heredia D.J., Smith T.K. (2012). Ca^2+^ transients in myenteric glial cells during the colonic migrating motor complex in the isolated murine large intestine. J. Physiol..

[54] Sharkey K.A. (2015). Emerging roles for enteric glia in gastrointestinal disorders. J. Clin. Investig..

[55] Gulbransen B.D., Sharkey K.A. (2009). Purinergic neuron-to-glia signaling in the enteric nervous system. Gastroenterology.

[56] Boesmans W., Martens M.A., Weltens N., Hao M.M., Tack J., Cirillo C., Vanden Berghe P. (2013). Imaging neuron-glia interactions in the enteric nervous system. Front. Cell. Neurosci..

[57] Gomes P., Chevalier J., Boesmans W., Roosen L., van den Abbeel V., Neunlist M., Tack J., Vanden Berghe P. (2009). ATP-dependent paracrine communication between enteric neurons and glia in a primary cell culture derived from embryonic mice. Neurogastroenterol. Motil..

[58] Delvalle N.M., Dharshika C., Morales-Soto W., Fried D.E., Gaudette L., Gulbransen B.D. (2018). Communication Between Enteric Neurons, Glia, and Nociceptors Underlies the Effects of Tachykinins on Neuroinflammation. Cell Mol. Gastroenterol. Hepatol..

[59] Fung C., Vanden Berghe P. (2020). Functional circuits and signal processing in the enteric nervous system. Cell Mol. Life Sci..

[60] Wang J., Dahl G. (2018). Pannexin1: A multifunction and multiconductance and/or permeability membrane channel. Am. J. Physiol. Cell Physiol..

[61] Hanstein R., Hanani M., Scemes E., Spray D.C. (2016). Glial pannexin1 contributes to tactile hypersensitivity in a mouse model of orofacial pain. Sci. Rep..

[62] Zhang Y., Chen K., Sloan S.A., Bennett M.L., Scholze A.R., O’Keeffe S., Phatnani H.P., Guarnieri P., Caneda C., Ruderisch N. (2014). An RNA-sequencing transcriptome and splicing database of glia, neurons, and vascular cells of the cerebral cortex. J. Neurosci..

[63] Hillen A.E.J., Burbach J.P.H., Hol E.M. (2018). Cell adhesion and matricellular support by astrocytes of the tripartite synapse. Prog. Neurobiol..

[64] Perry E.K., Lee M.L., Martin-Ruiz C.M., Court J.A., Volsen S.G., Merrit J., Folly E., Iversen P.E., Bauman M.L., Perry R.H. (2001). Cholinergic activity in autism: Abnormalities in the cerebral cortex and basal forebrain. Am. J. Psychiatry.

[65] Deutsch S.I., Urbano M.R., Neumann S.A., Burket J.A., Katz E. (2010). Cholinergic abnormalities in autism: Is there a rationale for selective nicotinic agonist interventions?. Clin. Neuropharmacol..

[66] Kemper T.L., Bauman M.L. (2002). Neuropathology of infantile autism. Mol. Psychiatry.

[67] Sokol D.K., Dunn D.W., Edwards-Brown M., Feinberg J. (2002). Hydrogen proton magnetic resonance spectroscopy in autism: Preliminary evidence of elevated choline/creatine ratio. J. Child. Neurol..

[68] Wenk G.L., Hauss-Wegrzyniak B. (1999). Altered cholinergic function in the basal forebrain of girls with Rett syndrome. Neuropediatrics.

[69] Barshack I., Fridman E., Goldberg I., Chowers Y., Kopolovic J. (2004). The loss of calretinin expression indicates aganglionosis in Hirschsprung’s disease. J. Clin. Pathol..

