# Supplementary material for: Quantitative Spatial Analysis of Neuroligin-3 mRNA Expression in the Enteric Nervous System Reveals a Potential Role in Neuronal–Glial Synapses and Reduced Expression in Nlgn3R451C Mice
Source: Biomolecules. 2023 Jun 30;13(7):1063. doi: 10.3390/biom13071063 (PMC10377306; doi:10.3390/biom13071063)
Supplement: Supplementary file 1 [file biomolecules-13-01063-s001.zip › biomolecules-2457717-supplementary.pdf]

biomolecules

MDPI

Supplementary Materials

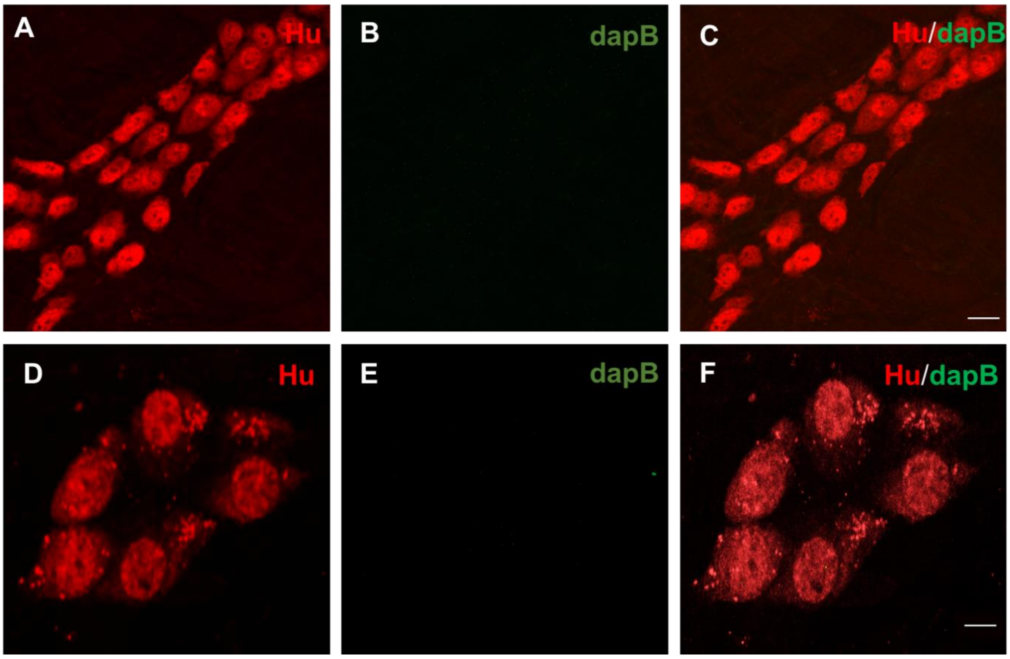

Figure S1. Negative control experiments for the myenteric plexus and the submucosal plexus. Myenteric neurons labelled with: **(A)** Hu pan neuronal marker and **(B)** the ACDBio RNAscope universal negative control probe (dapB) do not show detectable labelling. **C**: Merge. Submucosal neurons labelled with (**D**) Hu (**E**) dapB; F: merge. Scale bar=50µm.

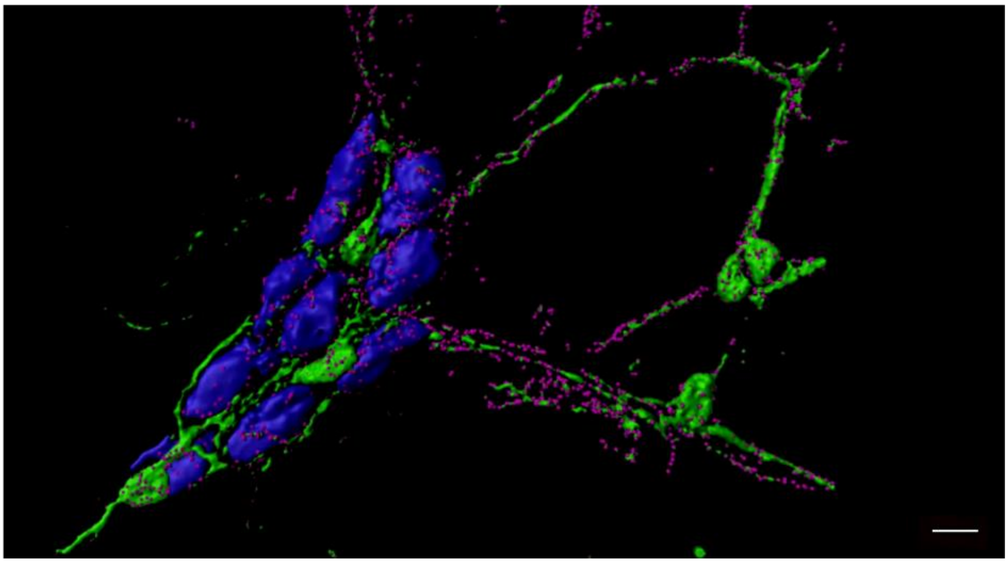

Figure S2. *Nlgn3* mRNA expression in submucosal neurons, glia and glial fibers

3D reconstructed image of a mouse ileal submucosal ganglion showing *Nlgn3* mRNA expression in glial fibers. Blue: Hu pan neuronal marker, green: S100 glial marker, magenta: *Nlgn3* mRNA, scale bar=50µm.

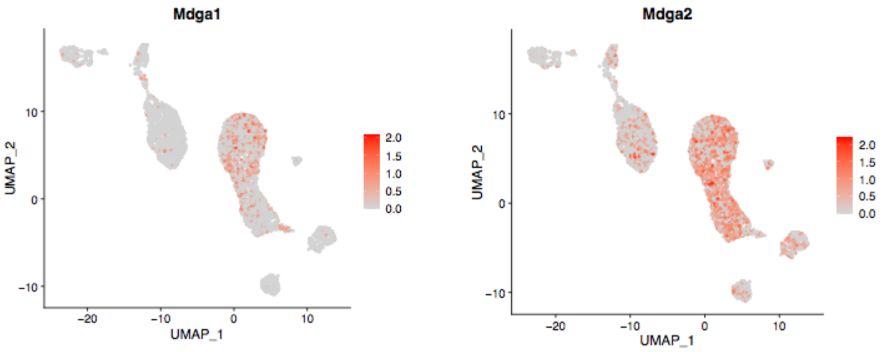

Figure S3. Expression of NLGN3 potential binding partners MDGA1 and 2 in mouse myenteric neurons. *Mgda1* was present in fewer than 50% of cells in ENC1 and 2 (excitatory motor neurons). *Mdga2* was expressed in a significant proportion of cells in ENC clusters 1-4 (excitatory motor neurons) and in a lower number of neurons belonging to clusters ENC8,9 (inhibitory motoneurons) and ENC12 (likely an heterogenous cell group). Scale bar refers to maximum and minimum expression levels within ENCs (refer to Figure 4 for legend).

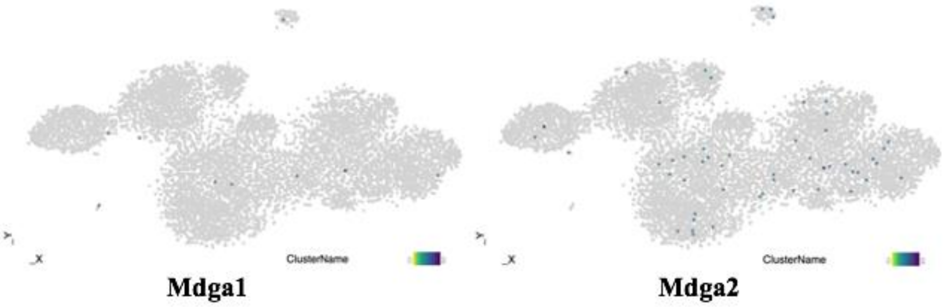

Figure S4. Expression of NLGN3 potential binding partners MDGA1 and 2 in mouse enteric glia. Scale bar refers to maximum and minimum expression levels within ENTGs and ENMFB (refer to Figure 5 for legend).

Table S1: Number of mice used for identifying enteric nervous system cellular subtypes. Where possible, the same mice were used to stain the submucosal and myenteric plexuses using immunofluorescence and RNAScope (total numbers of WT and *Nlgn3*^{R451C} mice used for immunofluorescence/RNAScope indicated in blue). For RNAScope and immunofluorescence experiments, a total of 34 WT mice and 29 *Nlgn3*^{R451C} mice were used. We also analysed historical RNASeq data from Mororach et al., 2021 (total of 5 WT mice).

Technique	Enteric neuronal plexus	Neurochemical marker	n (WT mice)	n (*Nlgn3*^{R451C} mice)
		Hu	14	10

**RNAScope and immunofluorescence (34 + 29 = 63 mice)**	Submucosal plexus	VIP	7	7
		ChAT	7	5
		S100b	6	5
	**Total samples**		**34**	**27**
	Myenteric plexus	Hu	14	10
		Calretinin	7	7
		NOS	7	7
		S100b	5	5
	**Total samples**		**33**	**29**
**RNAseq**<br>(Mororach et al., 2021)<br>**5 mice**	Myenteric Plexus	NA	5	NA
**Total mice**	**63 mice (RNAScope) + 5 mice (RNASeq) = 68 mice**			
